# Clinical characteristics, diagnosis, treatment, and prognosis of rituximab-induced serum sickness: a retrospective analysis of 39 reported cases

**DOI:** 10.3389/fimmu.2026.1798283

**Published:** 2026-04-13

**Authors:** Yi Huang, Wei Li, Yan Wang, Xiang Liu, Ying Huang

**Affiliations:** 1 Department of Pharmacy, The Central Hospital of Xiangtan (The affiliated hospital of Hunan University), Xiangtan, China; 2 Department of Pharmacy, Hunan University of Medicine General Hospital, Huaihua, China; 3 Department of Respiratory Medicine, Zhongshan Hospital of Traditional Chinese Medicine Affiliated to Guangzhou University of Chinese Medicine, Zhongshan, China

**Keywords:** anti-CD20 antibody, case reports, drug-related side effects and adverse reactions, rituximab, serum sickness

## Abstract

**Background:**

Rituximab-induced serum sickness (RISS) is an uncommon delayed hypersensitivity reaction with incompletely characterized clinical features and management. We aimed to synthesize published case reports/series to delineate clinical patterns, therapeutic strategies, and outcomes.

**Methods:**

PubMed, EMBASE, Web of Science, WanFang Data, and China National Knowledge Infrastructure (CNKI) were searched for rituximab-induced serum sickness reports published up to Nov 31, 2025, using keywords and free-text terms (e.g., “Rituximab,” “Serum Sickness,” “Serum Sickness-Like Reaction,” “Hypersensitivity,” “Adverse Drug Reaction,” “RISS,” and “anti-CD20”) with Boolean operators. Eligible case reports/series were screened and data were extracted with a standardized form. Study quality was evaluated using the JBI Critical Appraisal Checklist for Case Reports.

**Results:**

30 eligible articles identified 39 patients. The median age was 33 years (range 6, 86), with a female predominance (71.8%). The median symptom onset time was 7 days (range 1, 18) after last rituximab exposure. The most common indications were multiple sclerosis (23.1%), nephrotic syndrome (20.5%), and immune thrombocytopenia (20.5%). Clinically, arthralgia/arthritis (92.3%), fever (82.1%), and rash (66.7%) predominated. Anti-rituximab antibodies were positive in 90.9% of tested cases. Inflammatory markers were frequently elevated, with 93.3% of patients showing elevated erythrocyte sedimentation rate (ESR) and 91.3% showing elevated C-reactive protein (CRP). Complement consumption were frequent, with decreased C3 and C4 levels observed in 76.5% and 78.6% of tested patients, respectively. Corticosteroids were commonly used as the treatment for RISS, while switching to another anti-CD20 agent was primarily a strategy for managing the underlying disease. Overall, 82.1% achieved complete recovery and 15.4% improved, with a median recovery time of 3.0 days. Rechallenge was reported in 10 patients, with recurrence in 60.0%.

**Conclusion:**

RISS is a delayed reaction occurring after a free interval of several days, but it may clinically present shortly after a subsequent infusion in repeated dosing regimens. Its main features include the classic triad of fever, rash, and arthralgia or arthritis, commonly accompanied by elevated inflammatory markers and hypocomplementemia. Most patients improve rapidly after drug withdrawal, supportive care, and short-course corticosteroid therapy; however, rechallenge carries a substantial risk of recurrence and should be approached cautiously.

## Introduction

Rituximab is a chimeric anti-CD20 monoclonal antibody widely used in B cell malignancies and in a growing number of immune mediated diseases (1, 2). With broader indications and repeated exposure in routine practice, immune mediated adverse reactions have become an important clinical issue (3). Among them, rituximab induced serum sickness (RISS) is uncommon but clinically relevant because it can resemble infection or disease flare and occasionally cause significant systemic symptoms that require urgent management (4, 5).

Serum sickness is classically considered a type III hypersensitivity reaction driven by circulating immune complexes, complement activation, and systemic inflammation (6). Typical manifestations include fever, rash, and polyarthralgia or arthritis, usually occurring 1 to 2 weeks after the triggering exposure and potentially earlier after re-exposure (7). In rituximab-associated cases, anti-drug immune responses appear to contribute to pathogenesis, and anti-rituximab antibodies have been frequently detected in published reports (8). The clinical phenotype of RISS may overlap with other rituximab reactions, particularly acute infusion reactions or anaphylaxis (9). In contrast to immediate reactions that occur during or shortly after infusion, serum sickness typically presents in a delayed manner and is dominated by systemic inflammatory symptoms rather than bronchospasm and rapid cardiovascular collapse (10). Nevertheless, overlap has been described, underscoring the need for careful temporal assessment and differential diagnostic evaluation.

Several features make RISS challenging in routine practice. First, its presentation may be mistaken for intercurrent infection, autoimmune flare, or other drug reactions, prompting empiric antibiotics or extensive investigations (5). Severe cases of serum sickness can resemble sepsis, especially when hypotension or significantly elevated inflammatory markers are present (11). Second, the underlying disease appears to influence the risk. A study based on the French Pharmacovigilance Database suggested that rituximab-induced serum sickness may occur more frequently in patients with autoimmune diseases than in those with hematologic malignancies, highlighting the need for disease-specific vigilance (8). Third, the lack of a uniformly adopted diagnostic framework or biological evaluation for rituximab-induced serum sickness complicates the accurate assessment of this condition (12). Complement consumption and anti-rituximab antibody testing may support an immune complex process, but their availability and interpretation vary across clinical settings (13).

To facilitate earlier recognition and more consistent clinical decision-making, we performed a synthesis of published case reports and case series to characterize the clinical spectrum, management strategies, and outcomes of RISS. We specifically examined time to onset, laboratory profiles, recovery course, and recurrence following rechallenge. These findings aim to inform evidence-based recommendations for timely identification and optimized management of RISS in clinical practice.

## Methods

### Study design and literature search

We performed a synthesis of published case reports and case series describing RISS, including literature in English or Chinese. PubMed, EMBASE, Web of Science, WanFang Data, and China National Knowledge Infrastructure (CNKI) were searched from inception to Nov 31, 2025. We used controlled vocabulary (MeSH terms) and free text terms to identify relevant studies on rituximab exposure and serum sickness, including the following terms: “rituximab,” “anti-CD20,” “serum sickness,” “serum sickness-like reaction,” “hypersensitivity,” “adverse drug reaction,” and the following phrases: “rituximab-induced serum sickness” and “RISS,” using Boolean operators (AND/OR). References of eligible articles were also screened to identify additional reports.

### Inclusion and exclusion criteria

Reports were eligible if they (1) described patients who developed serum sickness or a serum sickness-like reaction attributed to rituximab, and (2) provided sufficient patient-level clinical information to support data extraction (for example, demographics, indication and dosing, onset latency, clinical manifestations, laboratory findings, treatment, and outcomes). Reviews, mechanistic or animal studies, duplicate cases, and reports lacking essential clinical details were excluded.

### Data extraction

Data were extracted using a predefined standardized form. The following variables were collected when available: age, sex, geographic region, indication for rituximab, comorbidities and relevant disease history, concomitant medications, rituximab dosing and exposure details, time to symptom onset, presenting features, key laboratory parameters (including anti-rituximab antibodies, inflammatory markers such as erythrocyte sedimentation rate (ESR), C-reactive protein (CRP), and complement levels, particularly C3 and C4), treatment measures (drug discontinuation, corticosteroids, antihistamines, epinephrine, immunomodulatory therapy, supportive care), clinical outcomes, time to recovery, and recurrence after rechallenge. Complete recovery is defined as the resolution of symptoms with no remaining signs of disease, while partial recovery refers to significant improvement in symptoms, but with some residual effects or ongoing management required.

### Quality assessment of case reports

The methodological quality of included case reports and case series was appraised using the Joanna Briggs Institute (JBI) Critical Appraisal Checklist for Case Reports (eight domains) (https://jbi.global/critical-appraisal-tools). Two reviewers independently rated each item as “Yes,” “No,” “Unclear,” or “Not applicable,” and disagreements were resolved by consultation with a third reviewer. Only studies meeting at least 70% of the JBI appraisal criteria were included.

### Causality assessment

Causality was classified using the WHO–UMC system (“certain,” “probable/likely,” “possible,” or “unlikely”). A “certain” classification required a clear temporal relationship, improvement after drug withdrawal, and recurrence on rechallenge. “Probable/likely” reflected a plausible temporal association with improvement after discontinuation without rechallenge. “Possible” cases had a reasonable temporal relationship but with competing explanations that could not be excluded. “Unlikely” cases lacked a supportive temporal pattern or were better explained by alternative causes.

### Statistical analysis

Descriptive analyses were performed. Continuous variables are presented as medians with ranges, and categorical variables as counts and percentages. Analyses were conducted using SPSS (version 23.0).

## Results

### Study selection

**Figure 1:**
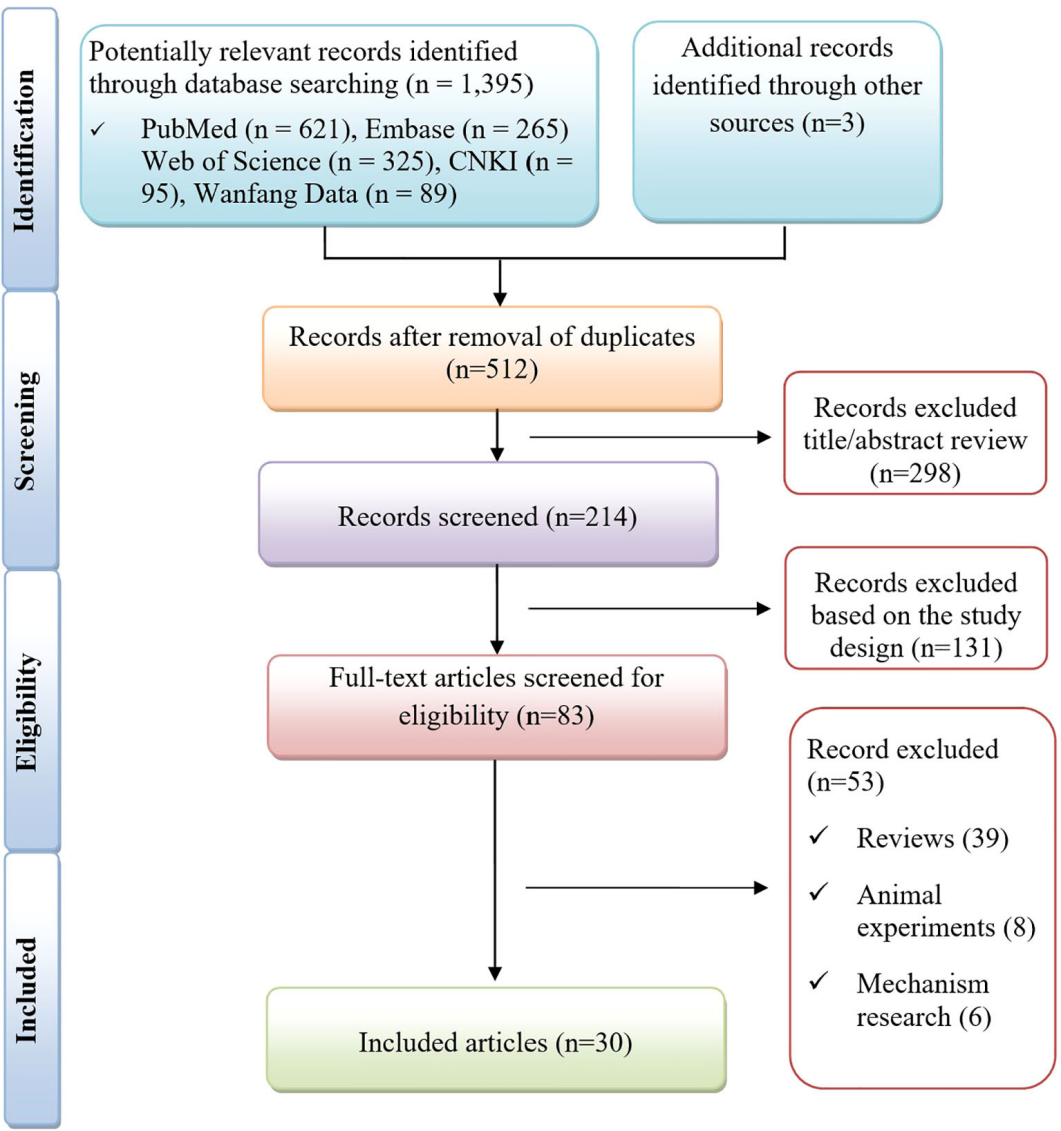
Flowchart illustrating the study selection process for inclusion.

1,395 records were identified through database searching and an additional three records were obtained from other sources (as shown in **Figure 1**). After duplicate removal, 512 records remained. Following preliminary exclusion, 214 records underwent title and abstract screening, of which 131 were excluded based on study design. Eighty-three full-text articles were then assessed for eligibility. Of these, 53 were excluded (reviews, animal experiments, and mechanism studies), leaving 30 eligible articles for final analysis (9–11, 14–40). These reports described 39 patients with RISS, and their baseline characteristics are summarized in **Supplementary Table 1**. Most cases clearly described patient characteristics, clinical manifestations, diagnostic evaluation, management, outcomes, and the main clinical takeaways. Item-level JBI assessments for each case are summarized in **Supplementary Table 2**.

### Basic information

As shown in **Table 1**, the overall cohort included 39 patients, with a clear female predominance (female 71.8%, n = 28; male 28.2%, n = 11). The median age was 33 years (range: 6–86). Cases were most frequently reported from the United States (30.8%, n = 12), followed by Japan (12.8%, n = 5), France (10.3%, n = 4), and Scandinavia (7.7%, n = 3); the remaining reports were distributed across China, Brazil, Canada, Iran (each 5.1%, n = 2), and other countries (17.8%, n = 7). The median symptom onset time after last rituximab exposure was 7 days (range: 1–18). Over half the cases occurred within 1–7 days (51.3%, n = 20), while 46.1% (n = 18) developed symptoms at 8–14 days; late onset (15–30 days) was uncommon (2.6%, n = 1). Rituximab was most commonly prescribed for multiple sclerosis (23.1%, n = 9), nephrotic syndrome (20.5%, n = 8), and immune thrombocytopenia (20.5%, n = 8), followed by lymphoma (10.3%, n = 4) and immunobullous disorders (7.7%, n = 3). Other indications accounted for 17.9% (n = 7). Rituximab dosing information was available for 26 patients. Among them, the most frequently reported regimen was 375 mg/m² (57.8%, n = 15), followed by 1000 mg (30.8%, n = 8). Less common doses included 500 mg, 250 mg/m², and 350 mg/m² (each 3.8%, n = 1). Concomitant medications were described in 13 patients, with clemastine (23.1%, n = 3) and azathioprine or tacrolimus (each 15.4%, n = 2) reported more often; other agents were each reported once.

**Table 1:** General data of 39 patients with rituximab-induced serum sickness.

Parameter	Classification	Value
Gender (39)^a^	Male	11 (28.2%)
Age (39)^a^	Years	33 (6, 86)b
Country (39)^a^	USA	12 (30.8%)
	Japan	5 (12.8%)
	France	4 (10.3%)
	Scandinavia	3 (7.7%)
	China	2 (5.1%)
	Brazil	2 (5.1%)
	Canada	2 (5.1%)
	Iran	2 (5.1%)
	Others (Israel, India, Italy, Qatar, Turkey, Uruguay, Spain)	7 (17.9%)
Time since last rituximab perfusion (39)^a^	Days	7 (1, 18)^b^
	1–7	20 (51.3%)
	8–14	18 (46.1%)
	15–30	1 (2.6%)
Indication (39)^a^	Multiple sclerosis	9 (23.1%)
	Nephrotic syndrome	8 (20.5%)
	Immune thrombocytopenia	8 (20.5%)
	Lymphoma	4 (10.3%)
	Immunobullous disorders	3 (7.7%)
	Others (IgG4-related disease, rheumatoid arthritis, HCV-related mixed cryoglobulinaemia, mixed connective tissue disease, Sjögren’s syndrome, refractory autoimmune polyneuropathy)	7 (17.9%)
Rituximab dose (26)^a^	250 mg/m²	1 (3.8%)
	350 mg/m²	1 (3.8%)
	375 mg/m²	15 (57.7%)
	500 mg	1 (3.8%)
	1000 mg	8 (30.8%)
Concomitant medications (13)^a^	Clemastine	3 (23.1%)
	Azathioprine	2 (15.4%)
	Tacrolimus	2 (15.4%)
	Romiplostim:	1 (7.7%)
	Methotrexate + hydroxychloroquine + leflunomide	1 (7.7%)
	Cyclosporine + cefepime	1 (7.7%)
	Mizoribine + mycophenolate mofetil	1 (7.7%)
	Imipenem + vancomycin	1 (7.7%)
	Bendamustine + ondansetron	1 (7.7%)

a Represents the number of patients with this parameter out of 39 patients.

b Median (minimum, maximum). HCV, hepatitis C virus; IgG4, immunoglobulin G4.

### Clinical manifestations and laboratory findings

Clinical presentations are summarized in **Table 2**. Arthralgia or arthritis was the predominant manifestation (92.3%), followed by fever (82.1%) and rash (66.7%). Edema occurred in 25.6%, and myalgia in 23.1%. Less common findings included conjunctival involvement (7.7%), hypotension or shock (5.1%), headache (5.1%), respiratory symptoms (5.1%), and gastrointestinal symptoms (5.1%). Isolated reports described sore throat and Kawasaki-like features. Anti-rituximab antibodies (ARA) were reported in 11 patients and were positive in 10 (90.9%). Inflammatory markers and complement levels were variably documented. Inflammatory markers were frequently elevated, with 93.3% of patients showing elevated ESR and 91.3% of patients showing elevated CRP. Complement consumption was also observed, with 76.5% of tested patients showing decreased C3 levels and 78.6% showing decreased C4 levels. Based on WHO–UMC causality assessment, 6 cases (15.4%) were classified as “certain,” 29 (74.4%) as “probable,” and 4 (10.3%) as “possible”.

**Table 2:** Clinical information of 39 included patients with rituximab-induced serum sickness.

Parameter	Classification	Value
Clinical symptoms (39)^a^	Arthralgia/arthritis	36 (92.3%)
	Fever	32 (82.1%)
	Rash	26 (66.7%)
	Edema	10 (25.6%)
	Myalgia	9 (23.1%)
	Conjunctival involvement	3 (7.7%)
	Hypotension/shock	2 (5.1%)
	Headache	2 (5.1%)
	Respiratory symptoms	2 (5.1%)
	GI symptoms	2 (5.1%)
	Sore throat	1 (2.6%)
	Kawasaki-like features	1 (2.6%)
Anti-rituximab antibodies (11)^a^	Positive	10 (90.9%)
ESR (15)^a^	Elevated (*Ref: 0-20 mm/h*)	14 (93.3%)
CRP (23)^a^	Elevated (*Ref: 0-10 mg/L*)	21 (91.3%)
C3 (17)^a^	Decreased (*Ref: 90-180 mg/dL*)	13 (76.5%)
C4 (14)^a^	Decreased (*Ref: 10-40 mg/dL*)	11 (78.6%)
WHO-UMC causality category (39)^b^	Certain	6 (15.4%)
	Probable	29 (74.4%)
	Possible	4 (10.3%)

a Represents the number of patients with this parameter out of 39 patients.

b Under the WHO-UMC system, a “certain” case shows a clear temporal relationship with drug use, improvement upon withdrawal, and recurrence upon rechallenge. A “probable” case has a reasonable time relationship, is unlikely explained by other causes, and improves after withdrawal without requiring rechallenge. A “possible” case has a reasonable time relationship but may also be due to other conditions, and the effect of withdrawal is unclear.

C3, complement component 3; C4, complement component 4; CRP, C-reactive protein; ESR, erythrocyte sedimentation rate; GI, gastrointestinal.

### Treatment and prognosis

Management strategies varied (**Table 3**), but systemic corticosteroids were the most frequently used intervention (74.4%, n = 29). Adjunctive symptomatic measures included antihistamines (15.4%, n = 6) and nonsteroidal anti-inflammatory drugs (NSAIDs)/analgesics (15.4%, n = 6). A small proportion required acute supportive therapy with epinephrine (7.7%, n = 3). intravenous immunoglobulin (IVIG) was used in 5.1% (n = 2). Switch to an alternative anti-CD20 agent (obinutuzumab/ocrelizumab/ofatumumab) was documented in 20.5% (n = 8). Overall outcomes were favorable. Complete recovery was noted in 32 patients (82.1%), and partial recovery in 6 patients (15.4%). Among 26 patients with available recovery-time data, the median time to recovery was 3.0 days (range: 0.1–14). Most recovered within 3 days (≤1 day: 19.2%, n = 5; 2–3 days: 42.4%, n = 11), while 38.4% required 4–14 days (4–7 days: 19.2%, n = 5; 8–14 days: 19.2%, n = 5). Rechallenge information was available for 10 patients. Recurrence occurred in 60.0% (n = 6), whereas 40.0% (n = 4) had no recurrence after re-exposure, indicating a substantial relapse risk when rituximab is reintroduced.

**Table 3:** Treatment and prognosis of 39 included patients with rituximab-induced serum sickness.

Parameter	Classification	Value
Treatment (39)^a^	Corticosteroids	29 (74.4%)
	Antihistamines	6 (15.4%)
	NSAIDs/analgesics	6 (15.4%)
	Epinephrine	3 (7.7%)
	IVIG	2 (5.1%)
Switch to alternative anti-CD20 (obinutuzumab/ocrelizumab/ofatumumab)	8 (20.5%)
Outcome (39)^a^	Complete recovery	32 (82.1%)
	Improvement	6 (15.4%)
	Not reported	1 (2.6%)
Recovery time (26)^a^	Days	3.0 (0.1, 14)^b^
	≤1	5 (19.2%)
	2–3	11 (42.4%)
	4–7	5 (19.2%)
	8–14	5 (19.2%)
Rechallenge (10)^a^	Relapse/recurrence	6 (60.0%)
	No recurrence	4 (40.0%)

a Represents the number of patients out of 39 in whom information regarding this particular parameter was provided.

b Median (minimum, maximum).

IVIG, intravenous immunoglobulin; NSAIDs, nonsteroidal anti-inflammatory drugs.

## Discussion

RISS is an uncommon but clinically important adverse reaction, classified as a type III, immune complex-mediated hypersensitivity response (41). Although the mechanism remains incompletely understood, it is generally hypothesized that rituximab’s immunogenicity leads to the formation of antibodies that bind rituximab and promote immune-complex formation. However, the nature of these antibodies and antigens may vary, including ARA or antibodies against B cell intracellular antigens. Complement activation and exaggerated inflammatory response likely contribute to the systemic clinical manifestations and laboratory findings commonly observed in clinical practice, such as elevated inflammatory markers and complement consumption (42).

Our case-report–based synthesis supports several practical insights for recognition and management. First, RISS is a delayed reaction due to its immunological mechanism. However, symptoms may appear during subsequent infusions (e.g., during the second or third rituximab infusion), even though the immune response was triggered by the first infusion. In our dataset, symptom onset clustered within the first two weeks after exposure, which is distinct from acute infusion reactions that typically occur during or shortly after administration. This timing difference is often the most useful diagnostic clue and may also explain why RISS is underrecognized: patients may present days after infusion, and the reaction can be misattributed to infection or relapse of the underlying disease unless recent rituximab exposure is actively considered (43). Second, the clinical phenotype remains anchored in the classic serum sickness constellation—fever, rash, and arthralgia/arthritis—yet the spectrum is broader. Rare cases presented with hemodynamic instability or multisystem manifestations. These cases should be interpreted cautiously, as some may indicate overlap between RISS and acute hypersensitivity or anaphylaxis-related features, such as hypotension or shock, rather than isolated classic serum sickness alone. Such variability can prompt extensive evaluations and delay appropriate attribution. This is particularly relevant in autoimmune conditions (e.g., nephrotic syndrome, immune thrombocytopenia, multiple sclerosis), where baseline inflammatory symptoms or disease flares can overlap with RISS (15). In this setting, the diagnostic approach should prioritize a careful temporal association with rituximab, a focused exclusion of infection and alternative inflammatory etiologies, and supportive laboratory testing rather than reliance on any single symptom (42, 44). Third, laboratory assessment can strengthen diagnostic confidence but should be interpreted as supportive. ARA was frequently positive among tested cases in our analysis, consistent with immune-complex biology and suggesting that anti-drug antibody testing may be informative when available. In practice, ARA can be measured using immunoassays such as ELISA or electrochemiluminescence platforms, and some reports also use human anti-chimeric antibody (HACA) testing. However, antibody results are not conclusive: assay performance can vary, and timing of sampling is critical, with the optimal testing window typically being 1–2 weeks after symptom onset. Complement testing—particularly low C4—also often supports the diagnosis, but complement levels can be influenced by underlying disease activity and concurrent conditions (8). Therefore, antibody and complement studies should be integrated with clinical timing, symptom patterns, and exclusion of urgent alternatives.

Risk context also warrants consideration. Our cases spanned diverse indications, with many occurring in immune-mediated diseases. This aligns with pharmacovigilance observations that RISS may be more frequent in autoimmune populations than in hematologic malignancies, potentially reflecting baseline immune dysregulation, altered immune-complex clearance, or hypergammaglobulinemia (8). Re-exposure is another clinically relevant factor: RISS can occur early in treatment courses and also after subsequent administrations, indicating that prior tolerance does not eliminate risk. Pediatric nephrotic syndrome reports further emphasize that school-age patients can develop RISS after limited courses, sometimes with long intervals between administrations that may obscure sensitization risk (10, 11).

Management in our dataset was consistent with the principles of immune-complex hypersensitivity, with the cornerstone being the withdrawal of the offending agent when feasible to eliminate the antigenic driver. Mild cases may improve with symptomatic measures such as antihistamines and NSAIDs/analgesics. For more severe disease such as high fever, disabling arthritis, extensive rash, or systemic compromise (significant impairment of physiological function leading to organ dysfunction), systemic corticosteroids were commonly used and generally associated with rapid clinical improvement. A key practical point is that while corticosteroid premedication is effective in mitigating acute infusion reactions, its role in preventing RISS remains uncertain. Currently, there is no study comparing patients with and without corticosteroid premedication that demonstrates a significant difference in the incidence of RISS. Rechallenge remains one of the most difficult decisions, especially considering rituximab is often used for refractory disease. Our findings, in line with the study by Bayer et al., suggest that recurrence after rechallenge is not uncommon, with a recurrence rate of 14.3%, supporting a cautious approach (8). When rituximab cannot be permanently avoided, strategies described in practice include slower infusion protocols, intensified peri-infusion immunosuppression, co-administration of prophylactic fluids (e.g., normal saline up to 500 mL/h) to dilute anti-CD20 blood concentration, or switching to alternative anti-CD20 agents (45). Switching is biologically plausible because structural differences may change immunogenic epitopes and decrease the likelihood of recurrent immune-complex reactions; however, cross-reactivity cannot be excluded, and even humanized or fully human antibodies may remain immunogenic in susceptible hosts. Accordingly, any re-exposure plan should be individualized, explicitly discussed with patients, and accompanied by close monitoring.

### Limitations of the study

This study has several limitations inherent to a case-report–based synthesis. First, publication bias is unavoidable, as unusual or severe presentations are more likely to be reported. Second, diagnostic evaluations were heterogeneous across reports, and key investigations were inconsistently performed and documented, including anti-drug antibody testing, complement dynamics, and standardized severity assessment. Third, follow-up information—particularly regarding outcomes after rechallenge or switching to alternative anti-CD20 agents—was variably reported, which limits definitive conclusions on long-term risk and optimal subsequent management.

## Conclusion

Our analysis provides clinically concrete insights into the mechanism, recognition, and management of rituximab-induced serum sickness. RISS should be considered when delayed systemic symptoms occur after rituximab exposure, especially when fever, rash, and arthralgia/arthritis occur together with inflammatory marker elevation and/or complement consumption. Although the optimal timing for recognizing and managing RISS remains unclear, early recognition and intervention, including discontinuation of rituximab and appropriately tailored anti-inflammatory treatment (e.g., corticosteroids or other immune-modulating therapies), may be associated with better outcomes. Given the risk of recurrence, rituximab rechallenge should be approached cautiously and individualized to clinical necessity.

## Data Availability

The original contributions presented in the study are included in the article/**Supplementary Material**. Further inquiries can be directed to the corresponding author.
