# Supplementary material for: Clinical characteristics, diagnosis, treatment, and prognosis of rituximab-induced serum sickness: a retrospective analysis of 39 reported cases
Source: Front Immunol. 2026 Apr 13;17:1798283. doi: 10.3389/fimmu.2026.1798283 (PMC13111249; doi:10.3389/fimmu.2026.1798283)
Supplement: Supplementary file 1 [file Supplementaryfile1.docx]

**Supplementary table 1.** **The basic information of 39 patients with rituximab-induced serum sickness.**

| **Reference** | **Country or region** | **Gender /Age** | **Indications for rituximab** | **Dosage** | **Symptom onset time (day)** | **Arthralgia/Arthritis** | **Fever** | **Rash** | **Anti-rituximab antibodies** | **ESR** | **CRP** | **Complement Levels** | **Treatment** | **Evolution** |
| --- | --- | --- | --- | --- | --- | --- | --- | --- | --- | --- | --- | --- | --- | --- |
| Blase et al. (2022) | USA | F/25 | ES | 375 mg/m² | 18 d post 5th dose | + | + | - | NA | NA | NA | C4↓ (C3 normal) | CS | Improved |
| Kumar et al. (2025) | USA | F/34 | IgG4-RD | NA | 1 d post 3th dose | + | - | + | NA | ↑ | ↑ | normal | CS | Resolved |
| Le Guenno et al. (2011) | France | F/31 | ITP | 375 mg/m² | 13 d post 1th dose | + | + | + | NA | NA | ↑ | C4↓ C3↓ | CS | Resolved |
| Cottu et al. (2023) | France | F/86 | wAIHA | 375 mg/m² | 5 d post 2th dose | + | + | + | + | NA | ↑ | C4↓ C3↓ | CS+Epi | Resolved |
| Herishanu et al. (2002) | Israel | F/48 | ITP | NA | 6 d post 2th dose | + | + | + | NA | NA | NA | NA | CS | Resolved |
| Succaria et al. (2016) | USA | F/33 | TTP | NA | 7 d post 1th dose | + | + | + | NA | NA | NA | NA | NA | NA |
| Kumar et al. (2012) | India | F/60 | RA | 1000 mg | 6 d post 1th dose | - | + | + | NA | ↑ | NA | NA | CS+Epi+AH | Resolved |
| Catuogno et al. (2005) | Italy | F/60 | HCV-MC | 375 mg/m² | 7 d post 1th dose | + | + | + | NA | ↑ | ↑ | C4↓ | CS+AH | Resolved |
| Mehsen et al. (2008) | France | F/30 | MCTD | NA | 13 d post 1th dose | + | - | + | NA | ↑ | ↑ | C4↓ C3↓ | AH | Resolved |
| Khatib et al. (2020) | Qatar | M/33 | PV | 1000 mg | 11 d post 2th dose | + | + | + | NA | NA | ↑ | C3↓ | CS | Resolved |
| Holmøy et al. (2019) #1 | Norway/Sweden | M/43 | MS | 1,000 mg | 10 d post 1th dose | + | + | - | + | ↑ | ↑ | NA | NA | Resolved |
| Holmøy et al. (2019) #2 | Norway/Sweden | F/23 | MS | 1,000 mg | 12 d post 1th dose | + | + | - | + | NA | ↑ | NA | NA | Resolved |
| Holmøy et al. (2019) #3 | Norway/Sweden | F/34 | MS | 1,000 mg | 10 d post 1th dose | + | - | - | + | ↑ | ↑ | NA | CS | Resolved |
| DeMonaco et al. (2007) | USA | F/47 | FL | 250 mg/m² | 7 d post 7th dose | + | + | - | - | ↑ | ↑ | NA | CS | Resolved |
| D’Arcy et al. (2001) | USA | M/45 | AIPN | NA | 7 d post 1th dose | + | + | - | + | ↑ | ↑ | C4↓ C3↓ | CS | Resolved |
| Wolf et al. (2019) #1 | USA | F/69 | MS | 1000 mg | 7 d post 1th dose | + | + | + | NA | NA | NA | C3↓ | CS | Improved |
| Wolf et al. (2019) #2 | USA | F/50 | MS | NA | 11 d post 1th dose | + | + | + | NA | NA | NA | NA | NA | Resolved |
| Wolf et al. (2019) #3 | USA | M/31 | MS | NA | 14 d post 1th dose | + | + | + | NA | NA | NA | NA | NA | Resolved |
| Wolf et al. (2019) #4 | USA | F/27 | MS | NA | 10 d post 1th dose | + | + | + | NA | NA | NA | C3↓ | CS | Improved |
| Wolf et al. (2019) #5 | USA | F/31 | MS | NA | 7 d post 1th dose | + | + | + | NA | Normal | Normal | C3↓ | CS | Improved |
| Goto et al. (2009) | Japan | M/8 | ITP | 375 mg/m² | 10 d post 2th dose | + | + | + | + | ↑ | ↑ | C4↓ C3↓ | CS | Resolved |
| Sato et al. (2020) | Japan | F/6 | FRNS | 375 mg/m² | 7 d post 2th dose | - | + | + | + | NA | ↑ | C3↓ | CS | Improved |
| Yang et al. (2024) #1 | China | M/8 | PNS | 375 mg/m² | 6 d post 2th dose | + | + | + | NA | ↑ | ↑ | NA | CS+AH | Improved |
| Yang et al. (2024) #2 | China | F/9 | PNS | 375 mg/m² | 11 d post 2th dose | + | + | + | NA | NA | ↑ | NA | NA | Improved |
| Hellerstedt et al. (2003) | USA | F/23 | AITP | NA | 1 d post 2th dose | + | + | + | NA | NA | Normal | Normal | NA | Resolved |
| Fajt et al. (2014) | USA | F/37 | MALT lymphoma | NA | 3 d post 2th dose | + | + | + | NA | NA | NA | C4↓ | CS+AH | Resolved |
| Finger et al. (2007) #1 | Brazil | F/38 | HGP | 1000 mg | 1 d post 2th dose | + | + | - | NA | ↑ | NA | NA | CS | Resolved |
| Finger et al. (2007) #2 | Brazil | F/45 | pSS | NA | 7 d post 1th dose | + | + | + | NA | ↑ | NA | NA | CS | Resolved |
| Fujinaga et al. (2025) | Japan | M/7 | SDNS | 375 mg/m² | 8 d post 3th dose | + | + | + | NA | NA | NA | NA | CS | Resolved |
| Joubert et al. (2021) | France | F/31 | MS | 1000 mg | 10 d post 1th dose | + | - | + | NA | NA | ↑ | Normal | CS | Resolved |
| Bhagirath et al. (2012) | Canada | F/45 | TTP | 375 mg/m² | 5 d post 1th dose | + | - | - | NA | NA | NA | NA | NA | Improved |
| Torun Bayram et al. (2020) | Turkey | M/6 | MN | 375 mg/m² | 8 d post 1th dose | + | - | + | NA | NA | ↑ | C4↓ C3↓ | CS+Epi | Resolved |
| Nakamura et al. (2020) | Japan | M/6 | SDNS | 375 mg/m² | 10 d post 4th dose | + | + | + | + | NA | ↑ | NA | NA | Resolved |
| Kimura et al. (2022) | Japan | F/10 | FRNS | 375 mg/m² | 7 d post 10th dose | + | + | - | + | NA | ↑ | NA | CS | Resolved |
| Disperati et al. (2007) | Canada | F/52 | FL | 375 mg/m² | 1 d post 2th dose | + | + | - | NA | ↑ | ↑ | C4↓ C3↓ | CS+AH | Resolved |
| Mauvezin et al. (2025) | Uruguay | M/7 | SDNS | 350 mg/m² | 13 d post 1th dose | + | + | - | NA | ↑ | ↑ | C4↓ C3↓ | NA | Resolved |
| Gheisari et al. (2024) #1 | Iran | F/42 | PV | 500 mg | 7 d post 2th dose | + | + | - | NA | NA | NA | NA | CS | Resolved |
| Gheisari et al. (2024) #2 | Iran | F/35 | MMP | NA | 1 wk post 1st RTX dose | + | + | + | NA | NA | NA | NA | CS | Improved |
| Navarro-Matilla et al. (2021) | Spain | M/69 | NMZL | 375 mg/m² | 1 d post 3th dose | + | - | - | + | NA | ↑ | NA | NA | Resolved |

+: positive; -: negative.

AH, antihistamines; AIPN, autoimmune polyneuropathy; AITP, autoimmune thrombocytopenia; CS, corticosteroids; CRP, C-reactive protein; Epi, epinephrine; ES, Evans syndrome; ESR, erythrocyte sedimentation rate; FL, follicular lymphoma; FRNS, frequently relapsing nephrotic syndrome; HCV-MC, hepatitis C virus–related mixed cryoglobulinaemia; HGP, hypergammaglobulinemic purpura; IgG4-RD, IgG4-related disease; ITP, immune thrombocytopenia; MALT, mucosa-associated lymphoid tissue; MCTD, mixed connective tissue disease; MMP, mucous membrane pemphigoid; MN, membranous nephropathy; MS, multiple sclerosis; NA, not available; NMZL, nodal marginal zone lymphoma; PNS, primary nephrotic syndrome; pSS, primary Sjögren’s syndrome; PV, pemphigus vulgaris; RA, rheumatoid arthritis; SDNS, steroid-dependent nephrotic syndrome; TTP, thrombotic thrombocytopenic purpura; wAIHA, warm autoimmune hemolytic anemia.
